# New Roles for Vitamin D Superagonists: From COVID to Cancer

**DOI:** 10.3389/fendo.2021.644298

**Published:** 2021-03-31

**Authors:** David J. Easty, Christine J. Farr, Bryan T. Hennessy

**Affiliations:** ^1^ Department of Medical Oncology, Our Lady of Lourdes Hospital, Drogheda, Ireland; ^2^ Department of Genetics, University of Cambridge, Cambridge, United Kingdom; ^3^ Department of Molecular Medicine, Royal College of Surgeons in Ireland, Dublin, Ireland; ^4^ Department of Oncology, Our Lady of Lourdes Hospital, Drogheda, Ireland

**Keywords:** COVID-19, pancreatic cancer, pancreatic stellate cell, superagonist, vitamin D, paricalcitol

## Abstract

Vitamin D is a potent steroid hormone that induces widespread changes in gene expression and controls key biological pathways. Here we review pathophysiology of vitamin D with particular reference to COVID-19 and pancreatic cancer. Utility as a therapeutic agent is limited by hypercalcemic effects and attempts to circumvent this problem have used vitamin D superagonists, with increased efficacy and reduced calcemic effect. A further caveat is that vitamin D mediates multiple diverse effects. Some of these (anti-fibrosis) are likely beneficial in patients with COVID-19 and pancreatic cancer, whereas others (reduced immunity), may be beneficial through attenuation of the cytokine storm in patients with advanced COVID-19, but detrimental in pancreatic cancer. Vitamin D superagonists represent an untapped resource for development of effective therapeutic agents. However, to be successful this approach will require agonists with high cell-tissue specificity.

## Introduction

Vitamin D is a steroid hormone with well-characterized effects on bone metabolism and calcium homeostasis. More recently, attention has focused upon non-classical effects, including important roles in regulation of the immune response and lung function and less well described effects in multiple additional tissues such as the cardiovascular system (1–3).

Vitamin D deficiency appears to be widespread globally, although significant data gaps exist for low-income countries (4, 5). Deficiency has been defined as serum calcidiol (25 OH vitamin D) less than 50nmol/L in the USA. Insufficiency occurs between 50-75 nmol/L. To reduce the risk of infectious disease, the US Endocrine Society recommended serum calcidiol should be above 75nmol/L (6). Approximately 40% of Europeans live with moderate deficiency (below the 50nmol/L cut off) and 5% of the US population have severe deficiency with serum levels less than 30nmol/L (5, 7). Serum calcidiol levels show seasonal variation and at the end of winter, 36% of young adults in the USA have insufficient (70 ± 25nmol/L) vitamin D (8).

In developed countries, deficiency appears more common within specific populations. In the UK this includes BAME (Black, Asian and Minority Ethnic), elderly and obese individuals (9, 10).

Low serum calcidiol has been linked to increased susceptibility to a variety of diseases, including renal, dermatological, cardiovascular and autoimmune disease, infections and cancer (11, 12). Evidence suggests that vitamin D may protect against COVID-19 infection and decrease severity of symptoms in patients hospitalized with severe viral pneumonia. This might involve vitamin D inhibition of the cytokine storm and anti-fibrotic effects in the acute respiratory distress syndrome (ARDS) responsible for much mortality in COVID-19 (3, 13, 14).

Here we briefly review the physiology of vitamin D and regulation of target gene transcription. We describe activated vitamin D receptor (VDR) binding to DNA elements within promoters of target genes in association with coactivators and corepressors. Next, we discuss vitamin D superagonist (VDSA) interaction with the normal process of transcription to supercharge target gene expression. Then, in the main part of this review, we describe the pathophysiology of vitamin D with particular reference to COVID-19 and pancreatic cancer. Finally, we discuss approaches for the development of therapeutically useful VDSAs in some common human diseases.

## Vitamin D Physiology

The physiology of vitamin D has been extensively reviewed (3, 15) and is only briefly considered here. Cholecalciferol is obtained from ultraviolet light-induced cutaneous synthesis and to a lesser extent from diet. Within the skin, cholesterol precursor 7-dehydrocholesterol is converted to vitamin D3, which is hydroxylated to 25(OH)-D3 (calcidiol) in the liver and then to the active metabolite, 1,25(OH)2-D3 (1α, 25-dihydroxycholecalciferol, (calcitriol) in the kidney. Importantly, conversion of calcidiol into calcitriol also occurs within multiple tissues including normal and malignant epithelial cells and activated macrophages, suggesting an autocrine/paracrine function in non-calcium regulating cells (15, 16).

Calcitriol binds to the vitamin D receptor (VDR), a nuclear hormone receptor and transcription factor, present in most human tissues (17). The VDR contains a ligand binding domain (LBD) comprising one β-sheet and 13 α-helical structures. Helix H-12 contains an activation function-2 (AF-2) domain, which forms a hydrophobic binding pocket for vitamin D and its analogues. Vitamin D binds the ligand-binding pocket (LBP) of VDR-LBD and triggers a conformational change resulting in dimerization, and co-activator and co-repressor binding. Activated VDRs bind to vitamin D receptor elements (VDRE) within promoter and enhancer regions of target genes to regulate gene expression. VDR-binding pattern within the genome and the identity and number of genes targeted is dependent upon specific cell type, with between 200-1000 vitamin D target genes per cell) (17, 18).

VDRs bind to VDREs as either homodimers or a heterodimer with the retinoid X receptor (RXR). Basal transcription factors also bind together with transcriptional coactivators, including: steroid receptor coactivator‐1 (SRC‐1), glucocorticoid receptor interacting protein‐1 (GRIP1), steroid receptor coactivator 1 (SRC-1), amplified in breast cancer 1 (AIB-1) and glucocorticoid receptor interacting protein (GRIP), see below). The VDR/RXR/VDRE complex modulates the epigenome *via* recruitment of coactivators that increase histone acetylation and upregulate transcription. VDR/RXR complexes also bind to VDRE in association with co-repressors to suppress transcription of vitamin D target genes. The VDR also interacts with chromatin modifying proteins including BRD7 and KDM6B (19). Vitamin D signaling results in up or down-regulation of gene expression involved in common cellular functions. This includes proliferation, differentiation, cell adhesion, apoptosis and autophagy and affects multiple key biological processes, such as fibrosis, inflammation and immunity which are commonly disrupted in disease (3, 20).

### Vitamin D and the Immune System

Vitamin D has multiple effects upon cellular and humoral immunity. On the one hand, vitamin D stimulates the innate immune system *via* modulating activity of Toll-Like Receptors (TLR). Calcitriol binds to VDRs in macrophages resulting in increased production of antimicrobial secreted peptides such as defensin and cathelicidin and CD14, a glycoprotein, co-receptor for TLRs (21). Here, Th1 cytokines promote a pro-inflammatory response. On the other hand, vitamin D down-regulates the adaptive immune response, which may have beneficial effects, safeguarding against autoimmunity (22) (see below). Calcitriol inhibits B-cell activation and promotes Treg cell activity leading to cytokine mediated activation of Th2 and suppression of Th1 cells (23). T cell activation requires induction of VDR expression which occurs downstream of T cell receptor (TCR) signaling and activation of the p38 map kinase pathway (24). Vitamin D influences neutrophil and macrophage function. Neutrophil activity is decreased (25), likely resulting in an over-all beneficial effect in patients with COVID-19 and PDAC. However, vitamin D increases formation of neutrophil extracellular traps (NET), meshes composed of DNA fibers, histones and proteolytic enzymes. It is postulated that NETs may result in detrimental effects in patients with COVID-19 and PDAC) (26, 27) (see below). Alternatively, another group report vitamin D reduced expression of proinflammatory cytokines and inhibited NET formation, an effect seen only at low dose (28). Divergent findings between these studies may reflect the animal model used and differences between doses. Vitamin D also influences macrophage function; an initial pro-inflammatory, anti-tumor M1 phenotype is converted into immune-suppressive, pro-tumor M2 macrophages.

Vitamin D signaling is controlled through a feedback mechanism. VDR activation up-regulates *CYP24A1* expression, encoding vitamin D3 24-hydroxylase an enzyme that inactivates calcidiol and calcitriol, in a reaction dependent upon cytochrome P450. This reaction occurs in the liver and kidneys and prevents toxicity at physiological levels of vitamin D (20, 29). VDRs also localize in the membrane and mediate non-genomic actions, with ligand-dependent effects on signal transduction, affecting kinase and phosphatase activity. Calcitriol interacts with membrane VDR (mVDR, also known as membrane-associated rapid response steroid-binding proteins (1,25-MARRS). mVDR signals *via* mitogen-activated protein kinase (MAPK) and cyclic AMP (cAMP). Nevertheless, many of these effects only occur at supra-physiological concentrations of calcitriol, and the significance of these pathways remains uncertain (18, 29).

### Vitamin D and Physical Activity

Many factors combine to regulate vitamin D levels (see below), and these may include several less well-known mechanisms. Data from the ARIC study (10,342 participants) suggest physical exercise is associated with increased serum calcidiol. Vitamin D deficiency (<20 ng/mL) was significantly reduced in individuals meeting levels of physical activity recommended by the American Heart Association (30).

Recent data suggest physical exercise increases immunity and decreases the risk of cancer. Leisure-time physical activity was associated with decreased rates in 13 out of 26 cancers studied, although, melanoma and prostate cancer were exceptions with increased risk (31). The effect in infectious disease is less certain, although, physical activity has also been suggested to offer benefit against COVID-19 (32, 33). The benefits of physical activity are likely multifactorial, but may include increased levels of vitamin D, however, it is difficult to rule out effects of reverse causation in such studies.

## Vitamin D Superagonists

More than 3000 vitamin D analogues (VAD) have been synthetized, most directly derived from calcitriol and containing a variety of modifications. The aim has been to produce analogues with enhanced VDR binding and increased stability to metabolism. The structure of these compounds has been described previously and a further detailed description is beyond the scope of this review (34). Structural modifications within vitamin D have typically been made in four sites: the side chain, A-ring, CD-ring and triene system. More recently non-steroidal vitamin D mimics have also been described (34). To date, there have been few comparative studies of vitamin D and VDSAs in clinical trials. Most data derive from *in vitro* studies and animal models. Vitamin D analogues have been characterized using *in vitro* assays, including VDR binding affinity, reporter gene assays, and cellular assays for anti-proliferative effects and enhanced differentiation. However, since reporter gene assays may be influenced by analogue uptake and metabolism, further analysis of VDAs in yeast or cell-free transcription may be required to demonstrate increased activity (35).

Vitamin D superagonists (VDSA) demonstrate a significantly increased activity compared to calcitriol. Increased physiological effects are seen in cellular assays for anti-proliferative effects and enhanced differentiation. VDSAs also increase transcriptional activity in assays using reporter gene inserted downstream of promoters containing VDRE. Mechanisms for superagonist properties may include: (1) enhanced VDR-RXR dimerization, (2) increased co-activator recruitment and (3) reduced sensitivity to metabolism (36, 37).

Various VDR coactivators have been described. These include VDR Interacting Protein (DRIP205), also known as MED1 (mediator of RNA polymerase II transcription subunit 1), which directly interacts with the receptor. Additional interactions occurring with coactivators: SRC-1, AIB-1 and GRIP, have been described (38). Interestingly, many analogues displaying selective co-activator recruitment also demonstrate an enhanced tissue selectivity for activity. Here, tissue specific effects may arise secondary to preferential VDA VDR/RXR complex binding to VDREs in promoters upstream of target gene, as has been suggested for the IP9 type of VDRE (37, 39).

X-ray crystallography studies of VDAs complexed with the VDR yielded mechanistic information useful for ligand design. Orientation within the LBP influences agonist activity, and compounds with 20-epi side chain modifications sit inside this pocket to promote coactivator binding, mediating transcription at 100-fold lower concentration than calcitriol (40). Various additional side chain modifications have also generated ligands with superagonist activity (34). Removal of C19 from within the A-ring resulted in 19-nor vitamin D compounds (including paricalcitol) with increased pro-differentiation and anti-proliferative activity on cancer cells and decreased calcemic activity.

Finally, C/D ring modified derivatives such as the 14-epi-analogs of calcitriol, including TX527 and TX522 (inecalcitol) also show markedly increased anti-proliferative effects in *in vitro* assays and lower calcemic effects compared with calcitriol. Increased activity was associated with a tighter association of these VDAs with coactivators SRC-1 and DRIP205, where a 10-fold lower dose of inecalcitol was required for VDR-coactivator interaction compared to calcitriol (40).

On the other hand, vitamin D signaling may also be enhanced by decreasing interaction of the VDR with corepressors, resulting in increased transcription. However, to date, VDAs inhibiting corepressor interactions have not been described (41).

Several VDAs with enhanced efficacy have been described. Structures containing fluorinated side chains are resistant to degradation. These compounds, including CD578, mediate increased VDR-coactivator binding and stronger pro-differentiation activity compared to vitamin D *in vitro*. Fluorination stabilizes H12 resulting in enhanced binding to SRC-1, for VDR/CD578 compared to VDR/calcitriol (42).

More recently, Corcoran et al. (43) also describe double-point modified VDAs, derived from calcitriol with superagonist activity. Compared to calcitriol, these compounds are less calcemic with lower toxicity (resulting in less weight loss in experimental animals) and mediate more than ten-fold increased pro-differentiation effects in keratinocyte (HaCat) and acute myeloid leukemia (HL60) cell lines.

Finally, a group in China reported the synthesis of novel VDR ligands with non-secosteroidal structures based upon a phenyl pyrrolyl pentane backbone. Preclinical studies suggest some of these (including compound I5) may be useful for treatment of patients with pancreatic cancer [(44); discussed below].

VDAs have also been identified using high throughput screening of chemical compound libraries and in silico screening methods (45, 46) assayed a set of 21 potential VDAs previously identified by high throughput screening of a 10K chemical compound library (the Tox21 qHTS data set). Interestingly, they found a wide range of structurally diverse chemicals displayed VDA activity. Most of these compounds induced VDR signaling *via* effects upon heterodimerization with RXRα and coactivator and corepressor recruitment (46).

## Pathology of Vitamin D

Traditionally recognized for its role in childhood rickets and adult osteomalacia, there is a growing recognition that vitamin D deficiency may confer increased risk in multiple additional conditions, ranging from COVID-19 to cancer (20, 47, 48). Several extra-skeletal roles for vitamin D are recognized, but this has resulted in only a small number of therapeutic options using vitamin D agonists. Patients with chronic renal failure and secondary hyperparathyroidism have been treated with paricalcitol (49) and plaque psoriasis with topical calcipotriol (50). Epidemiological data and preclinical studies suggest that vitamin D deficiency may play a role in multiple common diseases such as cancer, coronary artery disease, fibrosis and infectious and autoimmune diseases (12, 20). Hence there is much current interest regarding possible beneficial effects of vitamin D supplementation. In addition, a new idea is emerging that VDAs may offer new therapeutic avenues for several common diseases.

### Autoimmunity

Consistent with its role in inhibition of the acquired immune response, vitamin D seems likely to decrease risk of autoimmune diseases (including diabetes mellitus type 1 (51), multiple sclerosis (MS) and systemic lupus erythematosus) and immune mediated diseases such as inflammatory bowel disease (IBD). Epidemiological and preclinical data support a role for vitamin D in MS. Interestingly, calcitriol reduced demyelination in experimental autoimmune encephalomyelitis (EAM) a mouse model of MS. This was associated with increased Treg activity, activation of Th2 and suppression of Th1 cells (52). However, a therapeutic role in IBD and autoimmune diseases including MS has been limited due to hypercalcemia of vitamin D at doses required for treatment (20, 53).

### Musculoskeletal Conditions

Vitamin D protects against fracture risk through several mechanisms, including effects upon bone, muscle strength and immunoregulation. Multiple studies find a positive association between serum calcidiol and bone mineral density. Moreover, vitamin D directly affects muscle function to decrease risk of falls. Finally, osteoporosis appears to be initiated by pro-inflammatory cytokines, driving increased bone metabolism and vitamin D may down-regulate inflammation through effects upon the immune system thereby decreasing risk of fracture (54, 55).

Numerous epidemiological studies have analyzed serum calcidiol and risk of osteoporotic fractures. The data is conflicting with some studies showing support for a protective effect (56, 57). Consistent with this idea, several interventional trials of vitamin D indicate a reduced risk of fracture (54). Furthermore, a meta-analysis of supplementation (vitamin D plus calcium) versus fracture risk, found a significant reduction in total fractures and concluded vitamin D to be a useful preventative intervention for fracture risk reduction (58).

Finally, a recent meta-analysis of 41,738 patients studied the correlation between serum calcidiol and risk of senile osteoporotic fractures. High serum levels were associated with a reduced risk of hip fractures in elderly patients, but not with reduction in total fracture risk. The authors suggested that disparate results seen in earlier studies may have arisen due to selection of fracture sites studied and analysis of both perimenopausal and senile osteoporotic fractures (59).

### Fibrosis

Pathological fibrosis occurs in multiple diseases. Following repetitive injury there is a replacement of parenchyma by scar like tissue, resembling an unhealed wound. Occurring in tissues such as the liver (associated with alcohol and chronic viral infections) and kidney, fibrosis is driven by release of TGF-β from macrophages or damaged parenchymal cells, together with growth factors (such as CTGF and PDGF). These mediators activate signal transduction pathways in stromal cells and increase production of the extracellular matrix (ECM). TGF-β signaling mediates phosphorylation of SMAD2 and SMAD3. Then a SMAD2/3/4 complex translocates into the nucleus, binds to SMAD-binding elements and drives expression of pro-fibrotic genes (60).

The VDR directly interacts with SMAD3 and inhibits TGF-β-SMAD signal transduction, an effect that is independent of VDR-mediated transcription (60, 61) found calcitriol inhibited TGF-β upregulation of pro-fibrotic genes in mouse kidney epithelial cells, and reduced plasminogen activator inhibitor–1 and α-SMA expression. These same authors went on to synthesize two VDAs that inhibited TGF-β without activation of classical VDR gene expression. They found 1,25-lactone and two synthetic derivatives of 1,25-lactone (DLAMs) inhibited pro-fibrotic signals without hypercalcemia. X-ray crystallography found 1,25-lactone and DLAMs interaction with the H12 helix differed from calcitriol, providing a mechanistic explanation for their properties and a template for further attempts to design therapeutically useful new VDAs. The authors suggested that selective VDAs may prove useful for anti-fibrosis treatments (60, 61).

Work from Evans and colleagues found that quiescent hepatic stellate cells (HSC) rapidly expanded following tissue injury, resulting in fibrosis in murine liver. This response was inhibited by VDA calcipotriol (62). Interestingly, vitamin D receptor knock out (VDRKO) mice developed spontaneous liver fibrosis. Moreover, activation of VDR signaling inhibited TGFβ-SMAD-dependent transcription of pro-fibrotic genes in HSCs. The authors suggested VDR ligands might be a potential therapy in liver fibrosis (62). A similar effect was seen for pancreatic stellate cells (PSCs) with calcipotriol repression of chronic pancreatitis in a mouse model (63). Furthermore, a number of other groups have found VDA inhibition of fibrosis in additional tissues using animal models in heart, kidney and skin [reviewed in (13) and references within].

Pathological fibrosis occurs in multiple diseases including renal disease, colon and pancreatic cancer (associated with cancer associated fibroblasts (CAF) and in the lungs of patients with COVID-19 that develop ARDS (discussed below). Vitamin D inhibition of the TGF-β-SMAD signaling pathway may offer a useful therapeutic approach in such patients.

### Infectious Disease

It has been suggested that low vitamin D status confers an increased risk of viral respiratory infections, including influenza (20). Some preclinical studies support this idea. Incubation of primary human bronchial epithelial cells with calcitriol *in vitro* increased secretion of pro-inflammatory cytokines CXCL8 and CXCL10. Such cytokines might be expected to recruit macrophages and play a role in antiviral responses (64). A protective role for vitamin D supplementation in patients with respiratory infections still remains controversial. A meta-analysis found reduced risk of acute upper and lower respiratory tract infections after supplementation (65). A recent randomly controlled clinical trial found a positive effect for vitamin D in patients with influenza (66). However, some other studies found no significant effect, and in one clinical trial the duration of symptoms was increased compared to placebo controls [(67); reviewed in (68)]. Vitamin D inhibited lung fibrosis in several mouse models and may have therapeutic potential in idiopathic pulmonary fibrosis (2). Much current interest concerns the putative protective role of vitamin D in pathogenesis of COVID-19.

## COVID-19

COVID-19 (coronavirus disease 2019) is caused by severe acute respiratory syndrome coronavirus 2 (SARS-CoV-2). The virus is closely related to several bat coronaviruses and it seems likely COVID-19 began as a zoonotic disease, with subsequent development of transmission between humans. Following initial isolation of SARS-CoV-2 in Wuhan (China), COVID-19 has now become pandemic (69). Globally, on the 30^th^ October, 2020, there were 44,592,789 confirmed cases of COVID-19, including 1,175,553 deaths (The world health organization (WHO) interactive Dashboard COVID-19 web site. Available from: www.WHO.INT, 2020); meanwhile, interactive web-based methods continue to track the progress of COVID-19 (70).

The SARS-CoV-2 receptor is angiotensin-converting enzyme 2 (ACE2), mediating viral entry together with TMPRSS2 protease activity (71). Alveolar type II epithelial cells and enterocytes are primary targets for infection, and damage to heart, lung, liver and kidney (organs expressing ACE2) is largely responsible for mortality in patients with COVID-19 (72). The severity of COVID-19 corresponds to the degree of host immune response against the virus. Infection results in effects ranging from: asymptomatic to mild respiratory symptoms (most commonly), severe lung injury (viral pneumonia and ARDS), followed by septic shock, and multiple organ failure. Among patients presenting with COVID-19 in Wuhan (China), ARDS occurred in 42% of those with severe pneumonia, and in 61–81% of cases admitted into intensive care (73, 74); a separate Chinese study reported deaths of around 65% of patients with ARDS (75).

### Evidence for a Protective Role for Vitamin D in COVID-19

Evidence from multiple sources is accumulating to suggest a protective role for vitamin D against COVID-19 (48). Indirect evidence suggests populations predicted to be vitamin D insufficient/deficient have higher rates of infection and severity of COVID-19. This includes individuals with diabetes, hypertension and obesity, all associated with low vitamin D status and increased COVID-19 mortality (76). Serum calcidiol concentration is dependent upon solar irradiation. Hence mortality is increased in people of color, with more melanized skin and proportionally reduced rates of vitamin D synthesis. This includes BAME individuals in the UK and African Americans in the USA (9, 10). Consistent with this, a small study of 392 health workers in Birmingham, (UK), found increased rates of seroconversion in patients with BAME ethnicity was associated with deficiency (< 30 nmol/l) of vitamin D (77). Nevertheless, other possible explanations cannot be excluded, perhaps including differences in socioeconomic factors (78–80).

Also consistent with this idea, latitude has been associated with COVID-19 mortality, likely related to rate of UV mediated cutaneous synthesis of vitamin D (76). Moreover, a comparative study across 20 European nations found a significant negative correlation between mean vitamin D level and COVID-19 related mortality comparing countries (81). However, such ecological approaches have been criticized, since confounding factors (including local screening methods and detection of COVID-19 cases) will make analysis difficult (82).

Studies, linking vitamin D status and outcome in patients with COVID-19 are summarized in **Table 1**. Most studies have been retrospective with small patient numbers. Serum vitamin D was correlated with outcome (biochemical, imaging and clinical, depending upon the study) in patients with COVID-19. Both the time of testing of serum vitamin D (sometimes years before COVID-19 infection; see below) and the cut off for vitamin D insufficiency/deficiency varied between studies.

**Table 1:** Observational studies, linking vitamin D status with outcome (severity of disease and mortality) in patients with COVID-19.

Study	N	Design	Effect	Reference
Patients diagnosed with COVID-19 were investigated for serum calcidiol and CT Thorax	73	Retrospective, observational	Higher VD^a^ associated with reduced lung involvement and better outcome. VDD^b^ associated with increased risk of mortality.	(83)
Patients ≥65 years, COVID-19 positive. Groups: VDD (≤30 nmol/L) versus VD replete. Assessed for in-hospital mortality, requirement for NIV. Biochemistry and CT Thorax.	105	Retrospective, observational	COVID-19-positive arm had lower serum calcidiol compared with COVID-19-negative arm. Patient with VDD had increased incidence of NIV^c^ and high dependency unit admission.	(84)
Serum calcidiol versus positive SARS-CoV-2 result.	107	Retrospective, observational	Serum VD is significantly lower in SARS-CoV-2 positive patients	(85)
Serum calcidiol measured in patients on day of admission and 8 weeks post PCR diagnosis of COVID-19. Results compared to symptoms, CT Thorax, biochemistry.	109	Prospective, observational cohort study	VDD was common, and not an indicator of pathology seen in CT-scans, or severity of symptoms.	(86)
Serum calcidiol in patients hospitalized with COVID‐19 versus disease severity	134	Retrospective, observational	VDD is associated with greater disease severity	(87)
Patients diagnosed with COVID-19, investigated for serum calcidiol at first presentation versus severe disease (IMV^d^ or death).	185	Retrospective, observational	VDD (≤30 nmol/L) was associated with higher risk of severe disease.	(88)
Serum calcidiol concentration versus clinical outcome and mortality due to SARS-CoV-2 infection. Where VD < 75 nmol/L is insufficient	235	Cross-sectional analysis	Significant association between VD insufficiency and increased mortality.	(89)
Serum calcidiol in COVID-19 positive and negative group	347	Retrospective, observational	No significant difference between groups.	(90)
Serum calcidiol or calcitriol measured in the year prior to COVID-19 testing versus risk of positive test.	489	Retrospective, observational	VDD status was associated with increased COVID-19 risk.	(91)
A previous serum calcidiol level was compared to risk of SARS-CoV-2 infection and severity of disease. Where VD < 75 nmol/L is suboptimal.	7807	Retrospective, observational	Low Serum VD was associated with increased likelihood of COVID-19 infection and hospitalization.	(92)
Serum calcidiol concentration versus SARS-CoV-2 positivity.	190,000	Retrospective, observational	Serum VD concentration is inversely associated with SARS-CoV-2 positivity.	(78)
Baseline serum calcidiol versus COVID-19 mortality.	341,484	Retrospective, observational. UK Biobank study.	No association between VD concentration and risk of severe infection and mortality.	(93)
VDD patients diagnosed with COVID-19 received: standard dose cholecalciferol or high dose ergocalciferol for 5 days	4	Case series	high dose VD supplementation shortened length of stay, lowered oxygen requirement, and reduced inflammatory marker.	(94)
Serum calcidiol tested in controls versus patients diagnosed with COVID-19	145	Case control study	VDD increases risk for COVID-19, most clearly seen in severe infections.	(95)
Frail elderly patients, with COVID-19 infection. Received VD in 3 groups: (1) preceding and (2) post diagnosis or (3) no supplementation.	77	Quasi-experimental	Survival was increased in Group 1 with regular supplementation over the preceding year	(96)

^a^VD, vitamin D; ^b^VDD, VD deficiency; ^c^NIV, Non-invasive ventilation; ^d^IMV, invasive mechanical ventilation.

Most studies found low vitamin D status was associated with increased disease severity and risk of mortality; this was confirmed by a recent meta-analysis (97). On the other hand, some reports have found no evidence for a protective role for vitamin D (48, 86), and a large UK Biobank study (93).

Grant and McDonnell questioned whether the multivariate analysis in the UK Biobank study was over adjusted for confounding variables. Furthermore, they posited that protective effects may not have been seen in the study due to low serum vitamin D in the majority of participants (98). Finally, in this study, vitamin D was assayed 10-14 years prior to the COVID-19 pandemic. Hence it was questioned whether serum levels would remain unchanged and be a useful indicator for levels at the time of infection (99, 100). A further caveat is that acute inflammatory diseases (likely including COVID-19) may affect serum calcidiol levels. Hence measurements at time of diagnosis may be less reliable than seasonally adjusted samples (101).

A quasi-experimental study found that survival was increased in frail elderly patients where there had been regular supplementation of vitamin D in the year preceding COVID-19 infection (96). Finally, Entrenas Castillo and colleagues report a small, pilot clinical trial in patients with COVID-19, where high dose oral calcidiol reduced the requirement for admission into intensive care (102).

**Figure 1:**
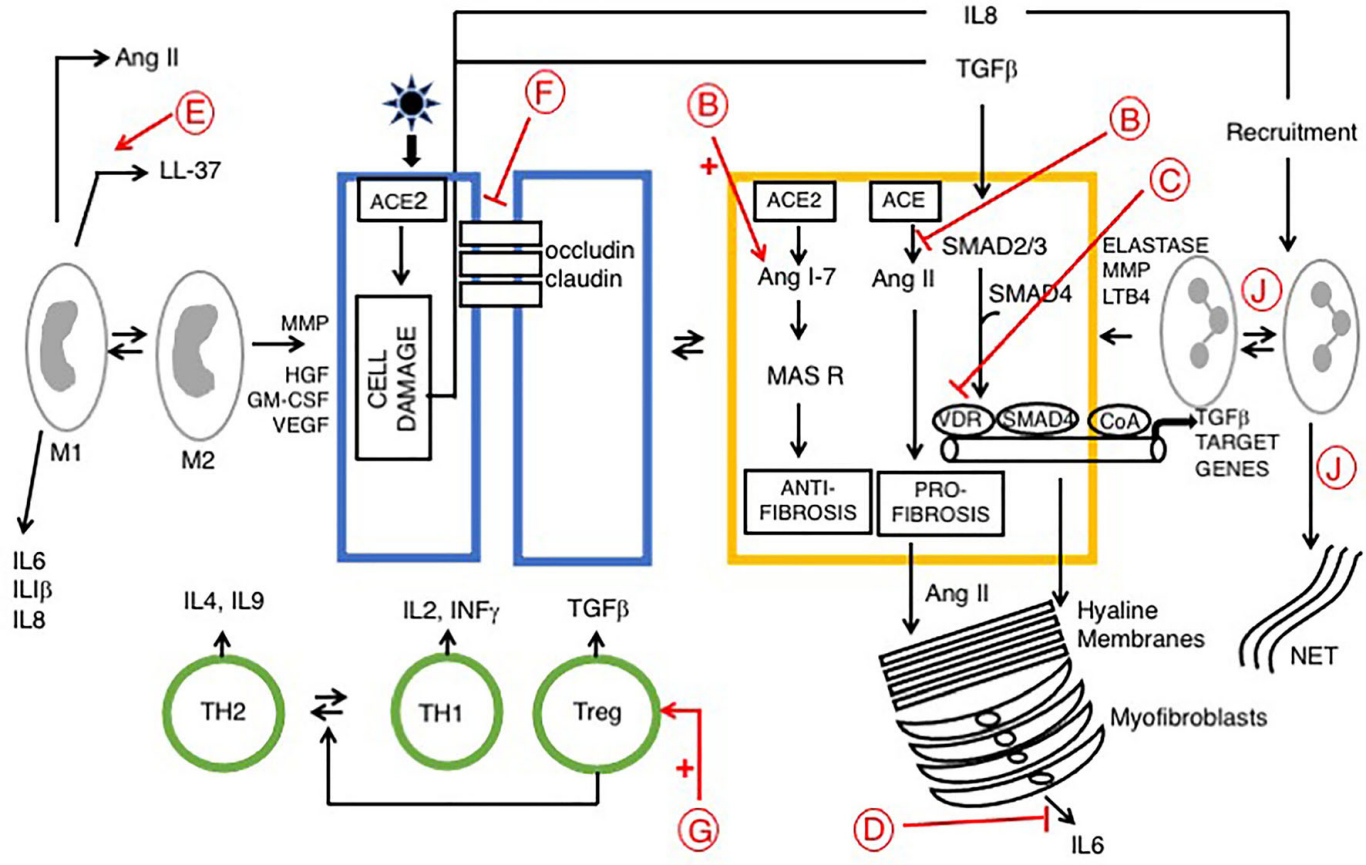
Signaling pathways in late phase ARDS in patients with COVID-19; role of vitamin D Disease progression is influenced by stromal/inflammatory infiltrate. Epithelial cells are colored blue, lung stellate cells (yellow), lymphocytes (green), and myeloid cells: macrophages M1, M2 and neutrophils (grey). SARS-CoV-2 infected alveolar type II epithelial (ATE2) cells release IL8, recruiting inflammatory cells, with neutrophil secretion of IL-6 contributing to the cytokine storm. Lung fibrosis occurs *via* two pathways. Macrophages and lung stellate cells secrete angiotensin II, promoting myofibroblast formation and increasing ECM formation with hyaline membrane disease. ATE2 cells secrete TGF-β, which drives the myofibroblast phenotype. Predicted effects of vitamin D are labeled in red A–J as described in **Table 6**, where labels indicate: B: Increased ACE/Ang II/AT1R signaling; C: Down-regulation of TGF-β-SMAD-dependent transcription; D: Inhibition of pro-inflammatory chemokine release; E: Increased antimicrobial peptides, defensins and cathelicidin; F: Maintains epithelial cell tight-junction integrity; G: Increased Treg activity; J: Decreased Neutrophil activity and increased NET formation. VDA effects D and E are likely more relevant in early stage COVID-19 respiratory tract infections.

Supplementation with vitamin D appears to protects against COVID-19. Counterintuitively, vitamin D increases ACE2 and facilitates SARS-CoV-2 entry. However, protective effects may be part explained by vitamin D stimulation of the innate immune system, with possible additional effects upon the renin–angiotensin system (RAS). SARS-CoV-2 binding to ACE2 drives an increase in ACE activity and angiotensin II production, resulting in vasoconstriction, pulmonary edema and increased severity of COVID-19. Calcitriol may play a protective role here *via* induction of ACE2 expression and inhibition of renin activity (103–105). Interactions between RAS and vitamin D in advanced COVID-19 are further discussed [see below and shown in **Figure 1** (function B)].

Taken together, the data strongly suggest vitamin D plays a protective role in patients with COVID infection and may decrease severity of ARDS. However, this hypothesis remains unconfirmed and data from a number of on-going clinical trials of vitamin D supplementation is eagerly awaited in order to better answer this question [**Table 2** and reviewed in (21)].

**Table 2:** Representative interventional clinical trials investigating Vitamin D in patients with COVID-19.

Study identifier:	Study design	^a^Dosage/Regimen/Route	N	Participants	Status
NCT04483635	Phase 3 Placebo-Controlled	^b^VD oral loading dose of 100,000 IU + 10000 IU VD weekly. Endpoint incidence of COVID-19 infection.	2414	^c^HCW caring for patients with COVID-19	Not yet recruiting
NCT04535791	Phase 3 Placebo-Controlled	VD, 4,000 IU orally daily for 30 days. Endpoint: COVID-19 infection status.	400	HCW caring for patients with COVID-19	Recruiting
NCT04536298	Phase 3 Placebo-Controlled	Daily VD for 4 weeks. Endpoints: hospitalization and/or death, risk of infection in household member.	2700	Newly diagnosed with COVID-19.	Not yet recruiting
NCT04386850	Phase 2/Phase 3 Placebo-Controlled	Calcidiol, 25 mcg once daily for 2 months. Endpoints: ARM 1 incidence of infection, severity of disease, hospitalization and death. ARM 2 severity and death.	1500	ARM 1 Prevention in HCWs. and ARM 2 Treatment of COVID-19 infected patients.	Recruiting
NCT04334005	Not Applicable	VD, 25000 UI, orally, daily. For 10 weeks. Endpoints: requirement for ^d^IAV, ^e^NIV and ^f^ICU admission.	200	Non-severe symptomatic, patients infected with COVID-19. Excludes patients presenting with ARDS.	Not yet recruiting
NCT04363840	Phase 2	VD, 50,000 IU, orally once weekly for 14 days + Aspirin 81mg, each day. Endpoint: Hospitalization.	1080	^g^VDD patients with new (24h) COVID-19 infection.	Not yet recruiting
NCT04385940	Phase 3	VD, 50,000 IU, orally, Determine low VD (<50 nmol/L), Endpoint: disease severity, Hospitalization.	64	VDD in inpatients/outpatients with COVID-19 infection.	Not yet recruiting
NCT04525820	Not Applicable Placebo-Controlled	Single high dose VD (140,000 IU) plus 800 IU of VD per day versus 800 IU of VD per day. Endpoint: Length of hospitalization until discharge or fatality	80	Hospitalized Patient Ongoing COVID-19 VDD	Not yet recruiting

^a^Dosage/Regimen in intervention group; ^b^VD, vitamin D; ^c^Health care workers (HCW); ^d^Invasive assisted ventilation (IAV); ^e^Non-invasive assisted ventilation (NAV); ^f^Intensive care unit (ICU). ^g^VDD, VD deficiency.

## Acute Respiratory Distress Syndrome

For the purpose of description ARDS may be separated into acute and late phases (106). Vitamin D may be detrimental in early COVID-19 due to attenuation of the antibody response. Conversely, it may be beneficial in patients with advanced COVID-19. Attenuation of the immune response likely prevents the cytokine storm and may decrease the severity of ARDS (13, 14). Here we consider predicted protective roles for vitamin D within each stage.

### Acute Phase

SARS-CoV-2 grows rapidly and damages lung tissue. During the first few days there is an influx of neutrophils and macrophages into the alveolar space resulting in alveolitis and loss of an intact alveolar epithelial barrier. This leads to intra alveolar edema and hyaline membranes are deposited onto basement membranes resulting in diffuse alveolar damage (DAD) characteristic of ARDS (107). Here, vitamin D delays pathology by decreasing neutrophil activity and maintains integrity of airway epithelial cell tight-junctions by up-regulation of occludin and claudin-5 in tight junctions. The inflammatory cell infiltrate produces proinflammatory cytokines and chemokines, constituting a cytokine storm that drives development of ARDS. Vitamin D protects against development of ARDS by inhibiting release of cytokines (including IL-6, IFN-γ, IL-1β) and chemokines such as CXCL8 and CXCL10 (21, 106). On the other hand, vitamin D increases NET formation and this has been suggested to contribute to severity of pulmonary inflammation (108).

### Late Phase

The late phase of ARDS (7 to 10 days after initial injury) is dominated by a fibro-proliferative process that fills the alveoli with granulation tissue, with increased ECM production and proliferation of myofibroblasts and type II alveolar cells. This is followed by irreversible pulmonary fibrosis (**Figure 1**). The renin‐angiotensin system (RAS) plays a role in development of ARDS. Two signaling pathways downstream of ACE and ACE2 play competing roles. The ACE2/Ang 1-7/MasR axis signals are anti-inflammatory and anti-fibrotic, whereas the ACE/Ang II/AT1R axis drives inflammation, fibrosis and vasoconstriction. ARDS pathology is driven by the second pathway, *via* unregulated Ang II, largely derived from lung fibroblasts and activated macrophages. Ang II then stimulates activation of lung myofibroblasts (109, 110). Vitamin D is protective, it reduces Ang II production and increases ACE2/Ang 1-7/MasR signaling, which may prevent the cytokine storm and inhibit development of ARDS (21). Evidence suggests that vitamin D deficiency may aggravate ARDS (111).

As discussed previously, VDAs inhibit TGFβ-SMAD-dependent transcription of pro-fibrotic and pro-inflammatory genes by transcriptional interference with Smad2/3 in the liver [(62); see above] and pancreas (63, 112) (see below). This same mechanism likely also operates in pulmonary disease, particularly given the recent identification of lung stellate cells, suggesting that anti-fibrotic agents such as paricalcitol may be beneficial in patients with ARDS (13).

Current clinical trials of vitamin D in patients with COVID-19 are summarized in **Table 2**. Most trials utilize oral vitamin D. Doses of vitamin D range from a single dose of 25,000 IU (NCT04334005) to 50,000 IU of vitamin D, once weekly for 2 weeks (NCT04363840). Liu et al. (113) suggested that a single large dose of vitamin D (300,000 IU) may be used for treatment of COVID-19. Finally, Evans and Lippman suggest that paricalcitol might be an effective treatment for patients with COVID-19 (13).

## Cancer

Vitamin D influences carcinogenesis *via* several mechanisms, including: (a) promotion of differentiation and inhibition of the epithelial mesenchymal transition, (b) regulation of cancer stem cells, (c) reprogramming of gene expression and induction of quiescence in cancer associated fibroblasts (CAFs), and (d) modulation of immune response (63, 114, 115). Observational studies suggest low vitamin D status is associated with an increased risk of cancer incidence and mortality (12, 116). Consistent with this idea, earlier studies found a significant inverse correlation between UVB irradiation and mortality rates for several cancers (117, 118).

### Cancer and Vitamin D Supplementation Studies

A large vitamin D intervention, random control clinical trial (VITAL) did not find a significant reduction in the primary end point of cancer mortality (119). Subsequent analysis found evidence for a protective effect; a second analysis of the data suggested decreased cancer mortality in some subgroups of enrolled individuals receiving vitamin D (18, 120). Very recently, a further analysis of the VITAL data suggests that vitamin D reduces the risk of metastatic or fatal cancer (121). Apparently consistent with this idea, a meta-analysis of 10 clinical trials (81362 pooled participants) found vitamin D supplementation was associated with a significantly lower risk of cancer mortality (47). Finally, a meta-analyses of clinical trials, testing high dose vitamin D supplementation (6537 participants), found a significant decrease in cancer mortality, although, there was no reduction in cancer incidence (122). Interestingly, several large scale studies, collectively indicate a greater effect upon progression rather than protection against carcinogenesis, consistent with previous reports (123).

Studies focusing on specific malignancies may provide a more homogenous data set for analysis. Here we describe vitamin D supplementation in preventative studies (breast and colon cancers and melanoma) and clinical studies in patients with advanced colon and pancreatic cancers.

A meta-analysis of vitamin D supplementation in breast cancer (72,275 participants) found relative risk reduction was below 30%, which lays within the futility boundary for the trial (124). On the other hand, two additional studies provide evidence suggesting vitamin D supplementation in healthy individuals inhibits carcinogenesis and mediates a reduction in breast cancer incidence (125, 126).

Epidemiological studies and clinical trials have investigated the role of serum calcidiol and vitamin D supplementation on the risk of colorectal cancer. Multiple prospective studies suggest vitamin D deficiency is a risk factor for colorectal cancer (127). On the other hand, vitamin D supplementation studies have yielded mixed results. No reduction of risk was observed in the Women’s Health Initiative, a trial in which 18,176 women received vitamin D and 18,106 received a placebo for an average of 7 years (128). However, the design of this study has been questioned regarding the low dose of vitamin D (400 IU/day) used for supplementation (129).

The role of vitamin D in melanoma is complex. A recent meta-analysis described a positive correlation between circulating calcidiol and risk of melanoma. Here ultraviolet B irradiation is a major risk factor and analysis of the relationship between serum calcidiol and melanoma is confounded by sun exposure. Importantly, no increased risk of melanoma occurred with vitamin D supplementation (130). Interestingly, several recent preclinical studies have suggested 7-dehydrocholesterol (a precursor of vitamin D) exerts anti-tumor activity in melanoma (131).

Extensive preclinical data from cell lines grown *in vitro* supports vitamin D anti-proliferative and pro-differentiation activity in multiple cancers, including prostate cancer and melanoma (132, 133); these effects occurred at supraphysiological concentrations of calcitriol (see below) (28).

Studies following the effect of vitamin D supplementation have also been conducted in patients with cancer: during surveillance of indolent prostate cancer and adjuvant treatment of colon and HER2+ breast cancer (134–137).

Interestingly, in the prostate study a second biopsy (performed after one year of vitamin D supplementation) found a decrease in number of positive cores or Gleason score in 55% of subjects; however, there was no significant change in PSA (134).

A small number of studies have evaluated vitamin D supplementation in patients with advanced cancer. The SUNSHINE, phase II clinical trial enrolled 139, untreated patients with colorectal cancer. Two groups received vitamin D (either 400 or 4,000 IU per day) in combination with best standard of care chemotherapy. This resulted in a median progression free survival of 13 months in the high-dose group, versus 11 months in the low-dose group (138). More definitive data are expected from on-going clinical trials.

Finally, vitamin D levels were measured in 1267 patients with PDAC. Pre-treatment serum, and OS were compared for patients with sufficient (>50 nmol/L), versus deficient (<25 nmol/L) levels. Survival was increased in patients with sufficient versus deficient serums in early stage PDAC, but not in patients with advanced stage PDAC (139). This interesting observational study requires further clinical trials to determine whether vitamin D supplementation might improve survival in patients with pancreatic cancer (see below).

The analysis of clinical studies of nutrients like vitamin D is complex and may require a different approach, compared to methods commonly used to follow pharmacological responses. Heaney focuses upon the importance of choosing a plausible dose range centered around where inadequate to adequate status occurs and suggests general guidelines for nutrient studies (140).

More recently, Boucher further explores Heaney’s ideas and additional factors complicating analysis of vitamin D supplementation studies (141); further confounding issues are also discussed elsewhere (18, 120, 121).

## Hypercalcemic Effect of VDR Ligands

Calcitriol has anti-tumor effects in animal models of various cancer types, and further, promotes the antitumor activities of various chemotherapeutic agents including 5FU, Gemcitabine and Paclitaxel (**Table 3**). However, this has not always been translated into clinical trials, and is thought to be largely due to hypercalcemic properties of calcitriol, limiting doses that can be delivered (154), but may also partly reflect deficiencies in trial design (155).

**Table 3:** Preclinical studies of VDA (Vitamin D Analogues) in animal models of various cancer types.

Tumor	Delivery/Dose	VD agonist	Effect	Reference
Prostate Ca, Dunning rat model.	s.c., 1mcg, 3x/week x3 weeks.	Calcitriol	Inhibition of tumor growth	(142)
Metastatic lung disease	s.c., continuous osmotic minipump rate 1 µg/kg/24 h x18 days 2.5 µl/h.	Calcitriol	Prevents met lung disease	(143)
Ovarian cancer, xenograft	gavage v placebo, 0.3 or 1.0 μg/kg body weight in a volume of 20μL, OD	Calcitriol	Suppression of growth	(144)
Breast cancer, nitrosomethylurea-induced rat mammary tumor model.	0.25 and 1.25 mcg/kg	Calcitriol	Inhibition of tumor growth, Hypercalcemia	(145)
Bladder cancer, xenograft	s.c., μg/mouse/d, x3 days GEM (6 mg/mouse/d, cisplatin (0.12 mg/mouse/d).	Calcitriol	Enhances activity of GEM and cisplatin	(146)
Pancreatic cancer xenograft	i.p., 2.5 and 5 mcg kg3×/week x28days	Calcitriol	Inhibition of tumor growth	(147)
Squamous cell Ca Xenograft	i.p., 80/160/320μg/mouse/day	Inecalcitol	Inhibition of tumor growth, increased apoptosis, decreased proliferation	(148)
Prostate Ca, xenograft	i.p.,1300μg/kg3×/week x42days	Inecalcitol	50% decrease tumor weight	(149)
Prostate Ca, xenograft	i.p.,0.5μg/kg every other day x45days	Seocalcitol	Reversal of growth stimulatory effects of PTHrP	(150)
Pancreatic cancer xenograft	s.c., 2.5μg/kg3×/week	Paricalcitol	Inhibition of tumor growth	(151)
Pancreatic cancer xenograft	i.p., 0.3μg/kg2×/week, x3weeks	MART-10	Inhibition of tumor growth	(152)
Breast cancer, nitrosomethylurea-induced rat mammary tumor model.	i.p.	Calcipotriol	Inhibition of tumor growth, No Hypercalcemia	(145)
UV-induced non-melanoma skin cancer	Topical application	Calcipotriol	Decrease in number and area of tumors combined with diclofenac	(153)
Pancreatic cancer, orthotopic model	i.p., 60 mg/kg QDX20, +/- GEM.	Calcipotriol	Induced stromal remodeling, increased intratumoral GEM, reduced tumor volume, increased survival.	(63)

Physiological levels of calcitriol in humans, range between 0.05–0.16nM. In animal models, anti-tumor activity of vitamin D requires supraphysiologic levels (156), and the therapeutic mechanism here is distinct from a simple correction of vitamin D deficiency. Supraphysiologic levels of calcitriol result in a limiting toxicity of hypercalcemia. This reduces dosage that can be administered to patients and is one possible explanation for the less marked anti-tumor effects seen in clinical trials (**Table 3**), as compared to the preclinical data (**Table 4**) (166).

**Table 4:** Completed Clinical trials of VDAs in various cancer types.

Tumor, Sample size	Delivery/Dose	Drug	Outcome	Reference
Prostate cancer n = 37	Rocaltrol (0.5mcg/kg) on day 1 + docetaxel (36 mg/m(2)) on day 2, repeated weekly for 6 weeks.	Calcitriol	30/37 (81%) achieved PSA response. 22 had 75% reduction in PSA.	(156)
Prostate cancer n = 250	DN-101, PO formulation, Weekly docetaxel 36 mg/m^2^ iv for 3 weeks of a 4-week cycle combined with either 45mcg DN-101 v placebo PO 1 day before docetaxel.	Calcitriol	Overall, PSA response rates were 63% (DN-101) and 52% (placebo), P = 0.07.	(157)
Prostate cancer n = 34	PO Dexamethasone, 1mg OD + 0.5mcg calcitriol at the start of week 5. Carboplatin (Area Under the Curve (AUC) = 2) started Week 7	Calcitriol	PSA response in 13 of 34 patients and had an acceptable side-effect profile	(158)
Prostate cancer n = 30	calcitriol 0.5 µg/kg PO, 4 divided doses over 4 h on day 1 + docetaxel 36 mg/m(2) i.v. on day 2 of each treatment week and zoledronic acid 4 mg i.v. on day 2 on the 1^st^ and 5^th^ week.	Calcitriol	PSA response in 47.8%	(159)
Metastatic NSCLC Phase I/II n = 34	Escalating doses: 30, 45, 60, and 80 mcg/m(2), calcitriol iv q21, prior to docetaxel 75 mg/m(2) and cisplatin 75 mg/m(2)	Calcitriol	Pre-specified endpoint 50% RR was not met	(160)
Pancreatic Cancer, non resectable n = 25	Calcitriol 0.5 u/kg on day 1, docetaxel 36 mg/m(2) IV on day 2, administered weekly for three consecutive weeks, and 1 week without treatment.	Calcitriol	Modest increase in TTP, 3/25 PR, 7/25 stable disease. Median TTP 15 weeks, and median OS 24 weeks.	(161)
Hepatocellular carcinoma n = 33	PO, 10μg/day, up to 1 year	Seocalcitol	2 complete response (CR), 12 stable disease (SD), 19 progressive disease (PD).	(162)
Pancreatic cancer n = 36	PO, 10–15μg/day x 8 week	Seocalcitol	No OR	(163)
Prostate cancer n = 54	PO, MTD 4mg/d + Docetaxel, max 18/52	Inecalcitol	85% response rate. As per PSA decline of 30%	(164)
Cutaneous metastatic breast cancer n = 19	Topical100μg/d, 6weeks	Calcipotriol	3 patients, 50% reduction in diameter of treated lesions	(165)
Metastatic Breast cancer n = 24	PO, 4–7 µg/day for 8 weeks + taxane	Paricalcitol	Most women tolerated 2–3mcg/d, (up to 7 µg per day without hypercalcemia	(151)

Administration of calcitriol orally (1.5-2.5mcg/day or 10.5-17.5mcg/week) was associated with a rate of hypercalcemia of 20-30% in prostate cancer patients (156, 167). VDSAs such as inecalcitol, which display up to 100-fold less hypercalcemic activity, have been developed in an attempt to circumvent this problem.

Paricalcitol demonstrates preclinical anti-tumor effects in various cancer types and clear anti-fibrotic activity. Reiter and colleagues found paricalcitol (but not calcitriol) inhibited fibrosis *in vivo* in the mouse CCl_4_ model, a finding likely of particular significance in PDAC (168).

### Pancreatic Ductal Adenocarcinoma

Pancreatic ductal adenocarcinoma (PDAC) has one of the poorest prognoses of all solid tumors. This likely reflects multiple factors, including desmoplasia. Desmoplasia is an increase in stromal cell proliferation of alpha smooth muscle (α-SMA) positive fibroblasts with increased extracellular matrix formation. Desmoplastic stroma restricts vasculature, impeding delivery of chemotherapy and inhibiting an immune response. In addition, reciprocal signaling pathways occur between PDAC and PSCs and most studies find activated PSCs facilitate PDAC growth (169, 170). Much interest surrounds targeting PSCs and HSCs in hepatic metastases from PDAC, the cells responsible for desmoplasia (171). Cancers arising within the pancreatic head compress the biliary outlet, decreasing absorption of fat soluble vitamins. The VDR is expressed by PSCs and PDAC cells. VDAs reduce PSC activation and are potential stromal targeting agents; there are also putative therapeutic effects upon malignant cells (172). Consistent with this, a genome-wide synthetic lethal screen identified VDR as one out of 27 validated genes that sensitized pancreatic cancer cells line (Panc1) to gemcitabine (173).

The VDR is expressed in PSCs, HSCs, and in cancer associated fibroblasts (CAFs) derived from these cells. VDR activation suppresses pro-fibrotic and pro-tumorigenic properties of cancer associated fibroblasts (CAFs). Moreover, spontaneous pancreatic fibrosis occurs in VDR knockout mice, consistent with a role for VDR inhibition of PSC activation (62). Importantly, calcitriol, the naturally occurring product of vitamin D metabolism and synthetic VDAs, inhibit tumor growth in xenografts of pancreatic cancer (63, 151). In addition, in preclinical studies, calcitriol potentiated cytotoxic activity of gemcitabine in human pancreatic cancer cell line (Capan-1) xenografts, with promotion of caspase dependent apoptosis (**Table 3**). Taken together, the data suggest that calcitriol exerts anti-fibrotic effects upon CAFs in pancreatic cancers. However, results from clinical trials with calcitriol have not generally demonstrated the activity expected from preclinical studies, in part because of dose limiting hypercalcemia (161).

A combination of Calcitriol and Docetaxel in PDAC resulted in a modest increase in time to progression, with 3/25 partial responses (PR) and 7/25 patients with stable disease (SD), (**Table 4**) (161). Recently, several new VDAs have emerged and clinical trial data suggests they may have anti-tumor effects in some specific cancer types.

VDSAs such as calcipotriol and inecalcitol (which display up to 100-200 fold less hypercalcemic activity) have been developed to circumvent the problem of dose limiting hypercalcemia (164, 174). Calcipotriol induced morphological changes in PSCs, with lipid droplet formation and decreased α-SMA expression. Calcipotriol mediated genome-wide changes in specific gene expression (with 664 increases and 1616 decreases), including down-regulation of IL-6, Stromal derived factor 1 (SDF-1) and collagen. Taken together, the data suggest that VDAs promote PSC differentiation and quiescence. In an orthotopic mouse model of PDAC, calcipotriol reduced desmoplasia and enhanced anti-tumor efficacy of gemcitabine resulting in a 58% increase in survival compared to gemcitabine alone (63). This important study became the basis for several clinical trials testing VDAs for anti-stromal activity in patients with PDAC (13).

A very recent study supports the data published by Evans’s group in 2014. Using a novel VDSA they replicate some of the original findings in an animal model of PDAC. Kang and colleagues synthesized 57 new non-secosteroidal VDR ligands (see above). Three of these compounds inhibited activation of PSCs, and in combination with gemcitabine, one of these (compound I5) demonstrated anti-tumor activity. In animal studies I5 activity displayed similar activity to calcipotriol with minor calcemic effects (44).

We previously planned a study testing inecalcitol (an orally bioavailable VDSA) in combination with standard of care gemcitabine/Nab-paclitaxel chemotherapy in patients with advanced PDAC. Unfortunately, inecalcitol became unavailable shortly prior to opening our Phase II clinical trial when Hybrigenics’ discontinued production in 2018. Most studies of VDAs in PDAC have utilized paricalcitol, which is currently the subject of several on-going clinical trials (**Table 5**), including our own recently opened Phase II study in patients with metastatic PDAC, (*ClinicalTrials.gov* identifier: NCT04617067).

**Table 5:** Completed and ongoing clinical trials involving Paricalcitol in patients with PDAC.

Study identifier:	Study design	Dosage/Regimen/Route	N	Patients	Status
NCT02030860	Phase I.	Paricalcitol (25mcg, IV), 3x weekly, with Nab-Pac and GEM D1, 8, 15, q 28 days.	15	Neoadjuvant and post-operatively.	Completed
NCT03520790	Phase I. Run-in safety study and Phase II Formulations (IV or Oral).	Paricalcitol (25mcg, IV), 3x per week, or PO OD, with Nab-Pac and GEM D1, 8, 15, q 28 days.	112	Metastatic	Recruiting
NCT03883919	Phase I.	Paricalcitol, 75 mcg IV on D1 and D8 combined with liposomal Irinotecan and 5-FU/LV.	20	Advanced PDAC, Progressed on GEM.	Recruiting
NCT03519308	Phase I.	Paricalcitol, and Nivolumab with Nab-Pac and GEM D1, 8, 15, q 28 days.	20	Resectable PDAC	Recruiting
NCT04054362	Phase II. PINBALL	Nab-Pac and GEM plus or minus cisplatin, followed by Paricalcitol, 25mcg PO (days 1, 3, 5, 8, 10, 12, 15 in a 28 day cycle)	14	Metastatic	Recruiting
NCT03331562	Phase II.	Pembrolizumab with paricalcitol (25mcg IV 3 xs per week) versus placebo.	24	Advanced PDAC	Recruiting
NCT03415854	Phase II.	Nab-Pac, GEM and Cisplatin. Plus paricalcitol Upon Disease Progression.	14	Metastatic	Recruiting
NCT03138720	Phase II	Paricalcitol with Nab Pac, GEM and cisplatin,	24	Neoadjuvant	Recruiting
NCT03300921	Phase Ib Pharmacodynamic Study.	Arm A: 50mcg IV weekly; Arm B: 12mcg PO once daily	20	Neoadjuvant	Recruiting
NCT02930902	Phase Ib	Arm A: Paricalcitol IV over D1, 8, and 15 and pembrolizumab IV d1, or Arm B: above, plus GEM and Nab-paclitaxel IV D1, 8, and 15.	23	Neoadjuvant	Recruiting

**Figure 2:**
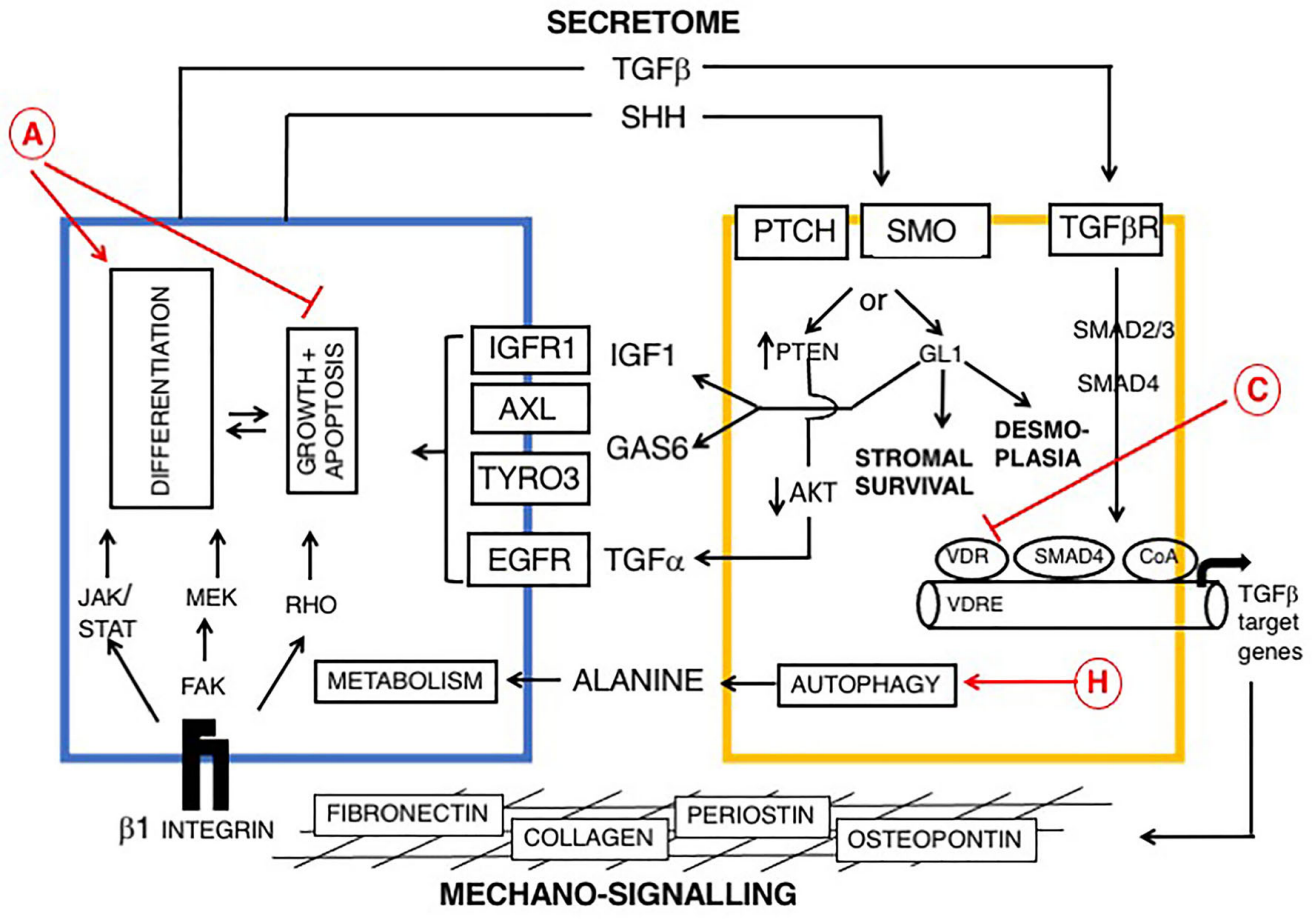
Reciprocal signaling pathways between PDAC and myofibroblasts (myCAFs) and role of vitamin D, modified from (169, 176, 177). Cells are colored as in **Figure 1**. Epithelial (PDAC) cells are blue and stromal cells, (PSCs) are yellow. Two broad pathways are shown: a PDAC-derived secretome, including TGF-β and Sonic hedgehog (SHH), upper and mechano-signaling *via* ECM/β-1 integrin and FAK (lower part of figure). TGF-β drives a myofibroblast phenotype. SHH signals *via* GLI to promote dysplasia. Ligands derived from myCAFs (including IGF1, AXL, TYRO3 and TGFα) support tumor growth and inhibit apoptosis. Predicted effects of vitamin D are labeled A-J as described in **Table 6**, where labels indicate: A Pro-Differentiation and Anti-Proliferation; C Inhibition of fibrosis and down-regulation of TGF-β-SMAD-dependent transcription of pro-fibrotic genes and H Promotion of autophagy in PDAC cells and CAFs with provision of alanine to PDAC cells (178, 179).

**Figure 3:**
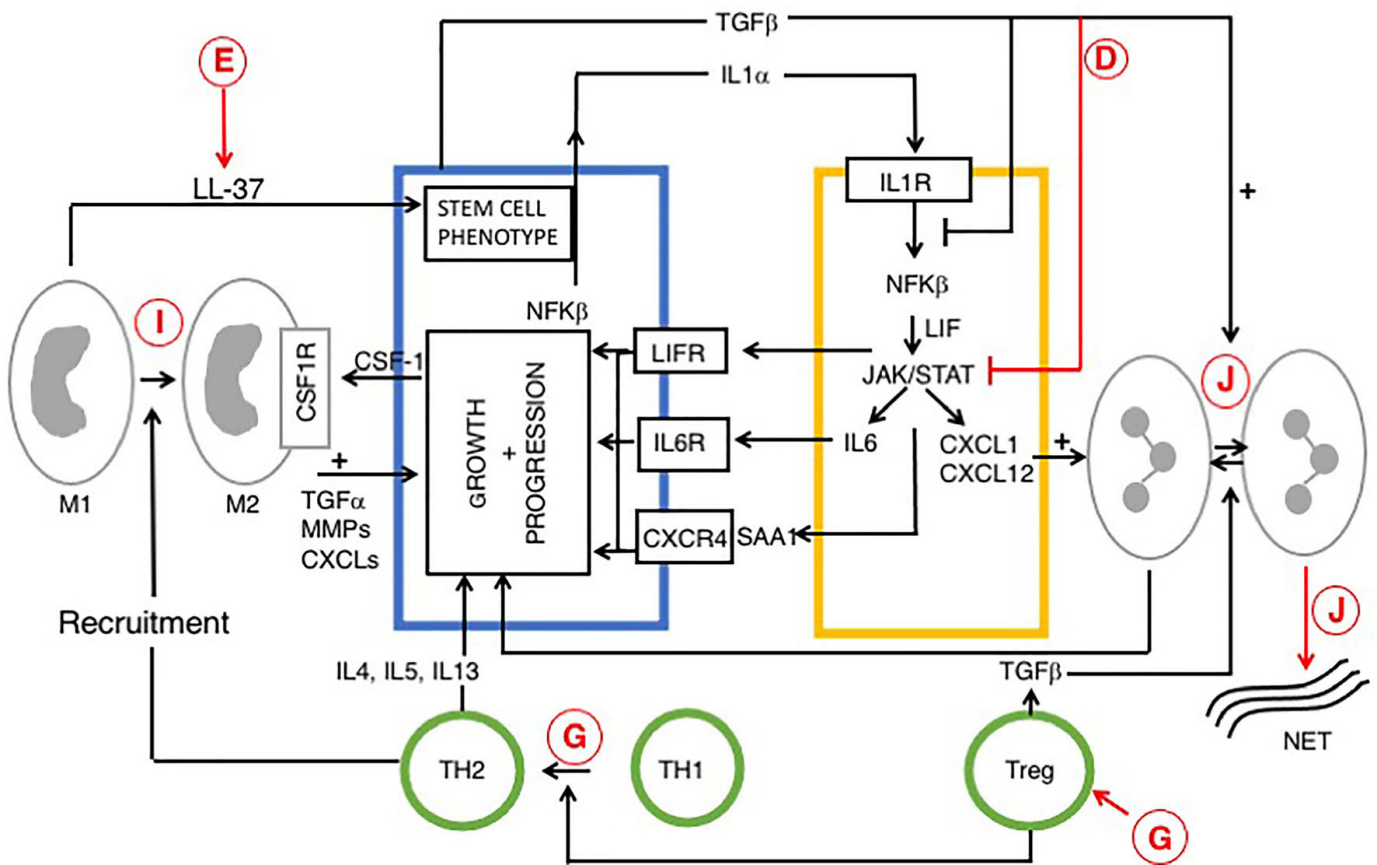
Reciprocal signaling pathways within PDAC and iCAFs, modified from (170, 180) and role of vitamin D. Tumor cell interactions with immune cells (tumor associated macrophages (TAM), neutrophils and T cells are also indicated. Cells are colored as in **Figures 1** and **2**. Stromal/inflammatory signaling pathways influence PDAC progression. Growth of PDAC cells is driven by cytokines and growth factors: LIF, IL-6 and SAA1 (derived from iCAFs), IL-4 (from TH2) and TGFα from TAMs. PDAC cells secrete IL-1α or TGF to drive an iCAF or myo-CAF phenotype. VDAs have direct effects upon cancer, stromal and immune cells and indirect effects upon intercellular signaling pathways. Predicted effects of vitamin D are labeled A-J as described in **Table 6**, where labels indicate: D Inhibition of cytokine and pro-inflammatory chemokine release; E Increased macrophage production of cathelicidin and cleavage product (LL-37), associated with a cancer stem cell phenotype; G Suppression of immunity: increased Treg activity, activation of Th2 and suppression of Th1 cells; I Anti-tumor M1 phenotype conversion into immune-suppressive, pro-tumor M2 macrophages, and J Decreased neutrophil activity and increased formation of NETs. The role of vitamin D in NET formation remains controversial and is discussed above. All figures, tables, and images will be published under a Creative Commons CC-BY license, and permission must be obtained for use of copyrighted material from other sources (including re-published/adapted/modified/partial figures and images from the internet). It is the responsibility of the authors to acquire the licenses, follow any citation instructions requested by third-party rights holders, and cover any supplementary charges.

Taken together, the data suggest that VDAs are likely to be useful stromal targeting agents in PDAC but this issue is complicated by stromal heterogeneity (175). Two subtypes of PSCs have been described. Inflammatory CAFs (iCAF) are α-SMA low/IL-6 high and express chemokines (CXC11 and CXC12), whereas myofibroblast CAFs (MyCAF) are contractile, stroma remodeling cells, with α-SMA high/IL-6 low. Reciprocal signaling pathways between PDACs and stromal cells (myoCAFs and iCAFs) are described in **Figures 2** and **3** respectively. Within unmanipulated surgical specimens iCAFs are located distantly from PDAC cells, whereas myCAFs were closely juxtaposed (181). Inhibition of iCAFs reduced PDAC volume in animal studies, *via* the IL1-α pathway (IL-1R, JAK/STAT, IL-6), suggesting these stromal cells are a useful target for stromal therapy (180). Blockade of IL-6 signaling from inflammatory CAFs may be a useful therapeutic avenue and results are awaited from a phase II study (NCT02767557), testing effects of Nab-paclitaxel and gemcitabine with Tocilizumab (an anti IL-6 receptor monoclonal antibody) in patients with PDAC.

Gene expression studies describe subtypes of PDAC, displaying distinct biological and clinical behaviors. Classical PDACs are well differentiated, respond better to 5FU based chemotherapy, and have a better prognosis than the poorly differentiated, basal-like subtype (182). Single cell sequencing studies of organoids established from PDAC biopsies found mixtures of classical and basal subtype cells coexisting within a single tumor. Where this is the case, chemotherapy might select for resistant subpopulations of PDAC cells and a shift in subtype may be seen during treatment (183).

Induction of differentiation of myCAFs together with inhibition of cytokine signaling in iCAFs might increase therapeutic effects of chemotherapy. A combination of VDA and a neutralizing antibody targeting signals from iCAFGs (anti IL-6R or LIF) might improve response to current best standard of care chemotherapy.

VDAs such as paricalcitol and calcipotriol mediate global changes in gene expression and affect multiple pathways (summarized in **Table 6**), including: (1) anti-stromal effects, (2) direct anti-proliferative and pro-differentiation effects on PDAC cells, and (3) effects upon the immune system.

**Table 6:** Predicted effects of VDAs in PDAC and COVID-19.

	Vitamin D/VDA effect	PDAC	COVID-19 (ARDS)	Reference
A	Pro-Differentiation/ Anti-Proliferation	Beneficial^a^	Uncertain	(172, 184)
B	Increases ACE2/Ang 1-7/MasR axis, inhibits ACE/Ang II signaling.	Uncertain	Beneficial	(109, 110, 185)
C	Inhibition of Fibrosis. Down-regulates TGFβ-SMAD-dependent transcription of pro-fibrotic genes.	Beneficial	Beneficial	(62, 63)
D	Inhibition of cytokine and pro-inflammatory chemokine release.	Beneficial	Beneficial	(63, 186)
E	Increases macrophage production of antimicrobial peptides, defensins and cathelicidin	Detrimental^b^	Beneficial	(187, 188)
F	Maintains integrity of epithelial cell tight-junctions	Uncertain	Beneficial	(21)
G	Suppresses immunity: increases Treg activity, activation of Th2 and suppression of Th1 cells	Detrimental	Beneficial	(52, 187)
H	Promotes Autophagy	Uncertain^c^	Uncertain	(178, 179, 189)
I	Pro-inflammatory, anti-tumor M1 phenotype is converted into immune-suppressive, pro-tumor M2 macrophages	Uncertain^d^	Uncertain	(75, 190)
J	Decreased Neutrophil activity	Beneficial^e^	Beneficial^e^	(21, 191)

^a^VDAs promote cancer cell and PSC differentiation. ^b^Cathelicidin active cleavage product (LL-37) is associated with PDAC stem cell growth and survival. ^c^Autophagy occurs in CAFs and resultant amino acids (including alanine) are made available to neighboring PDAC cells. Autophagy is likely a pro-survival mechanism in PDAC cells; ^d^M2 polarization may contribute to fibroproliferative phase of ARDS. ^e^Decreased neutrophil activation is expected to be beneficial; however, the role of vitamin D in NET formation remains controversial.

Vitamin D results in multiple effects, some of which may be detrimental in patients with advanced PDAC. A more recent study found that calcipotriol inhibited CAF proliferation and reduced secretion of pro-tumorigenic factors PGE2, leukemia inhibitory factor (LIF) and IL-6, consistent with the known anti-tumor effects of VDAs (187). This was also consistent with earlier studies that had shown LIF is secreted by PSCs, drives tumor progression and may be a useful therapeutic target in patients with PDAC (170). However, a new finding was that calcipotriol reduced CD8+T cell proliferation, decreased IFN-γ and IL-17, and increased IL-10 secretion, indicating an immunosuppressive effect (187). Furthermore, the authors discussed the role of vitamin D inducible peptide cathelicidin and its active cleavage product (LL-37) in PDAC. Previous work has shown that LL-37 is associated with cancer stem cell growth and survival in PDAC (188). Finally, vitamin D increases NET formation and recent work suggests this enhances hepatic micrometastasis in patients with PDAC (192).

Paricalcitol mediates beneficial effects upon the immune system. A pilot study of neoadjuvant paricalcitol in patients with resectable PDAC found 10-100 fold increased T cell migration into tumors after 28 days of treatment (193). Furthermore, a number of reviews suggest utilization of VDAs to inhibit immune-suppressive activity of stromal cells and the hope is that this will improve response to checkpoint inhibitors in patients with PDAC (194, 195).

Clinical trials using paricalcitol will likely benefit most from anti-stromal effects and subsequent improvement in delivery of chemotherapy. Taken together, the data suggest that overall, VDAs like calcipotriol decrease pro-inflammatory activity in patients with PDAC (187). Thus, VDSAs in clinical trials are predicted to result in significant benefits derived from decreased inflammation and desmoplasia but also with detrimental effects upon the immune system. It may be that selective agonists with reduced activation of Treg cells, while retaining strong inhibition of cytokine (IL-6) release will be particularly beneficial in patients with pancreatic cancer (**Table 6**; **Figure 3**).

PDAC subtype is influenced by intrinsic (genetics and epigenetics) and extrinsic factors, including chemotherapy and the stromal/inflammatory infiltrate. Inhibition of TAM and neutrophil activities result in a shift from basal to classical subtype. Finally, current interest surrounds the role of super enhancers in PDAC. In preclinical studies inhibition of BET family members resulted in a shift from basal to classical subtype, likely involving down-regulation of P63 (196, 197). It may be that BET inhibitors will sensitize basal subtype tumors to combined anti-stromal therapy and chemotherapy, suggesting new therapeutic approaches.

## Discussion and Perspectives

There is a growing recognition for the importance of extra-skeletal roles of vitamin D. Several key biological processes controlled by vitamin D are disrupted in common diseases, and it has long been anticipated this might provide new therapeutic targets. Efforts to target vitamin D signaling pathways have focused upon (a) correction of deficiency as a prophylactic measure to decrease disease severity, and (b) use of VDAs as therapeutic agents in advanced disease. It is instructive to compare the role of vitamin D signaling agents in patients with PDAC or COVID-19 (**Table 6**). Most current trials in COVID-19 have addressed early disease, whereas studies in patients with PDAC have focused on the neoadjuvant or metastatic setting and premalignant disease has not been targeted.

Epidemiological data suggest that vitamin D deficiency is common and may play a role in multiple diseases including cardiovascular and autoimmune disease (11); Fan, 2020 #276}. Multiple studies have addressed the role of vitamin D supplementation in extra-skeletal disease. Some, but not all, studies found positive effects for supplementation in respiratory infections including influenza (66, 68). Interpretation in such studies, is complicated by disagreement concerning serum levels of calcidiol required for sufficiency (198), multiple confounding factors (18, 120, 121) and the application of methods designed for testing drugs rather than nutrients (140, 141).

Several on-going clinical trials in patients with COVID-19 address early-stage disease and prevention of infection by vitamin D (NCT04483635 and NCT04535791), other studies have started patients onto vitamin D supplementation immediately after COVID-19 diagnosis (NCT04536298 and NCT04536298). No current study begins intervention in patients with COVID-19 following development of ARDs. Although most of these clinical trials have utilized vitamin D; one Iranian study (NCT04386850) used calcidiol (reviewed in (21)). Abnormal liver function tests (LFTs) are seen in 14–53% of patients with COVID-19, perhaps reflecting expression of ACE2 in cholangiocytes (199). Calcidiol seems a good choice for supplementation, since it does not require activation in the liver; calcitriol might also be an effective agent. Finally, Evans and Lippman suggest that paricalcitol might be a useful agent for future clinical trials in COVID-19 patients (13).

In the face of new emerging viral pathogens there will inevitably be periods without effective treatment in the time before vaccines can be prepared. The availability of VDSAs with anti-fibrotic activity may provide a useful approach in patients with ARDS secondary to influenza, COVID-19, or some as yet unknown, new viral respiratory tract infection.

Clinical trials of VDAs in pancreatic cancer have focused on advanced stage disease. Premalignant lesions are commonly found in patients prior to the development of PDAC. This includes pancreatic intraepithelial neoplasia (PanIN) seen early during PDAC development in both human pancreas and genetically engineered mouse models. Premalignant lesions are commonly present in patients with chronic pancreatitis with a 15-fold increased risk of PDAC (when present for more than 5 years) (200) and hereditary pancreatitis syndrome, where risk is increased by >25-fold (201). Stromal activation and ECM deposition occurs surrounding PanINs and it may be that prophylactic treatment with vitamin D or VDAs would reduce the incidence of pancreatic cancer development within these high-risk patients.

New tools have recently become available for interpreting the effects of stromal targeted therapy in PDAC. Firstly, gene expression studies have identified subtypes of PDAC and stromal cells displaying distinct biological and clinical behaviors, and secondly, organoids, a game-changing new technology which allow analysis of reciprocal signaling pathways between cancer and stromal cells. It may be that a combination of VDSA and Tocilizumab (an IL-6 receptor monoclonal antibody) targeting MyCAF and iCAFs will provide a useful adjuvant to current best standard of care chemotherapy in patients with PDAC.

Results from clinical trials testing vitamin D have often found decreased activity compared to prior expectations based upon preclinical studies. This has stimulated attempts to discover VDSAs with increased efficacy and reduced calcemic effect. A second aim has been to uncover agonists with high cell-tissue specificity. The search for new VDSAs has used high throughput screening of chemical libraries and computational modeling based methods. High throughput screens are often dependent on the promoters used to drive reporter gene readout and the cell lines used for the assay. Hirschfeld and colleagues placed a partial promoter sequence from the α-SMA gene upstream of a green fluorescent reporter protein, to specify myofibroblast function (202). The reporter gene was expressed within a rat hepatic stellate cell line and this system might provide a valuable assay for a high throughput screening to find VDAs with anti-fibrotic activity. Vitamin D likely promotes a mixture of beneficial and detrimental effects in any particular disease and consideration of **Table 6** suggests that selective agonists driving specific pathways may be most useful for treatment. Finally, selection of VDAs displaying decreased up-regulation of CYP24A1 expression may result in increased activity.

A large body of evidence suggests that vitamin D and VDAs likely possess therapeutic potential in several common diseases. We might soon anticipate a better understanding of their role in cancer and infectious disease. Two sets of clinical trials, comprising 42 and 11 studies in patients with COVID-19 and PDAC respectively, will deliver data within the next 2-3 years. The hope is that, where such studies yield positive results, this will act as a springboard to encourage isolation of further effective VDSAs. Selective VDAs represent an untapped resource for development of effective therapeutic agents. They may be useful in multiple diseases ranging from ARDS in patients with COVID-19 to tumor growth and metastasis in patients with pancreatic cancer.

## Author Contributions

DE and CF developed the idea for the review. All authors contributed to writing the manuscript and approved the submitted version.

## Conflict of Interest

The authors declare that the research was conducted in the absence of any commercial or financial relationships that could be construed as a potential conflict of interest.
